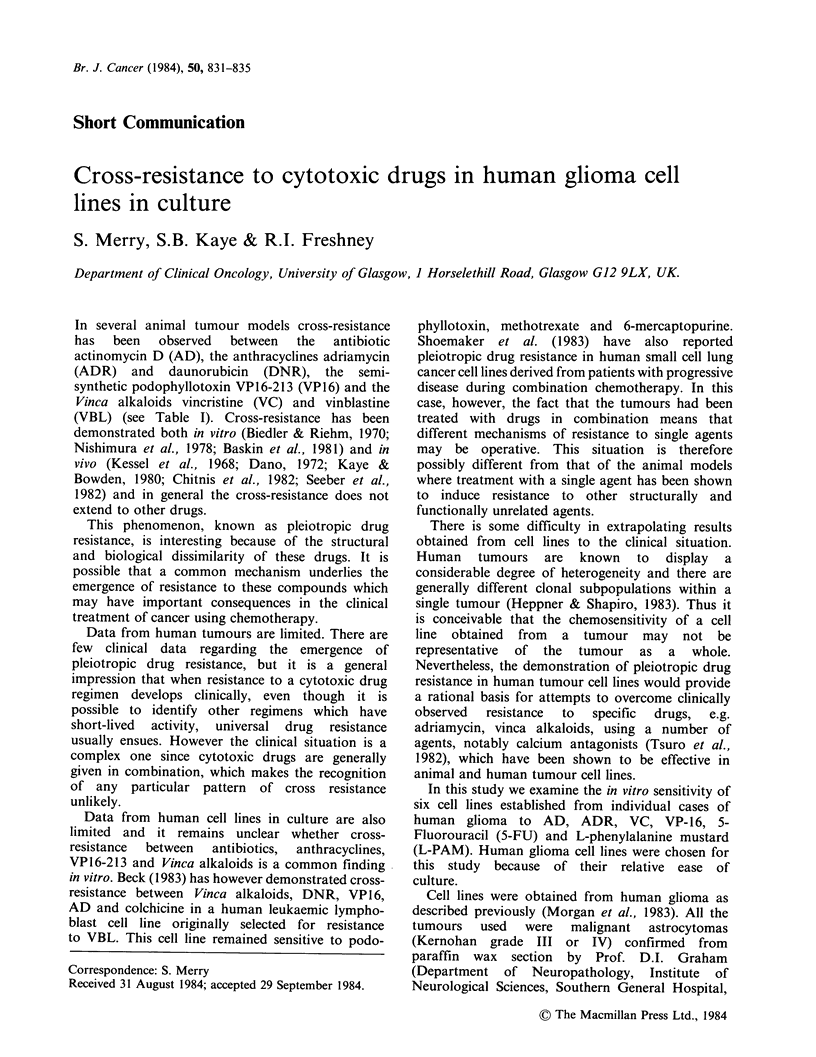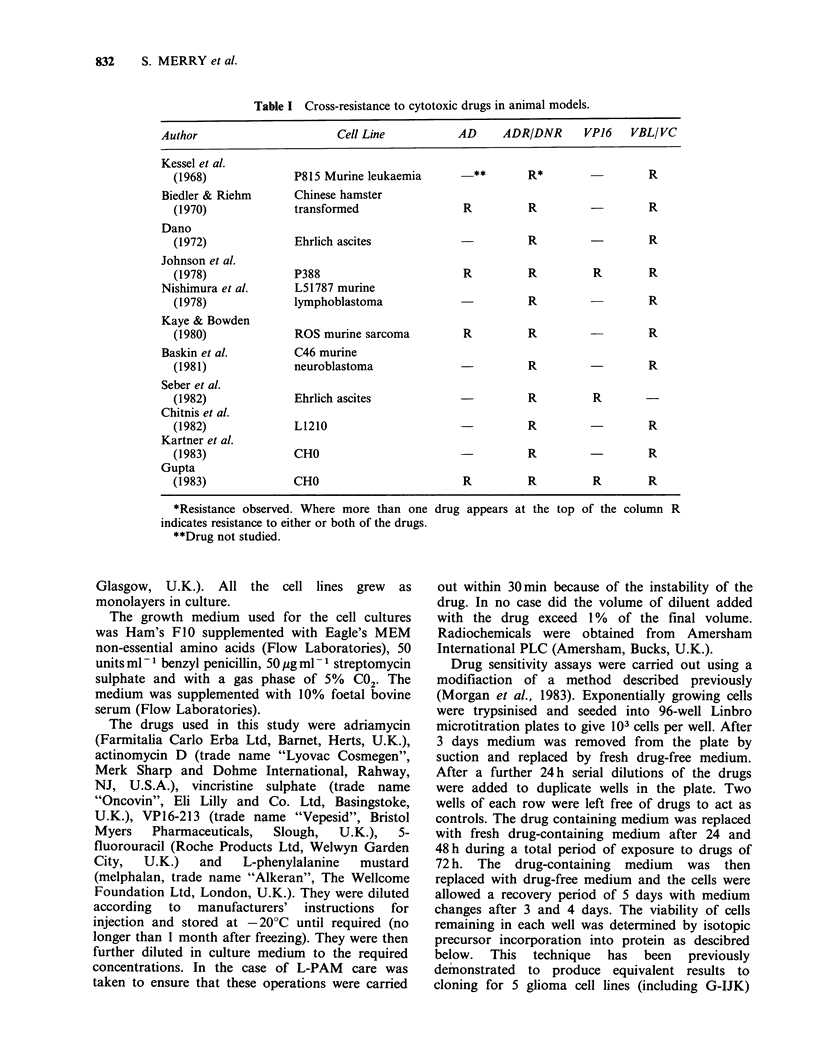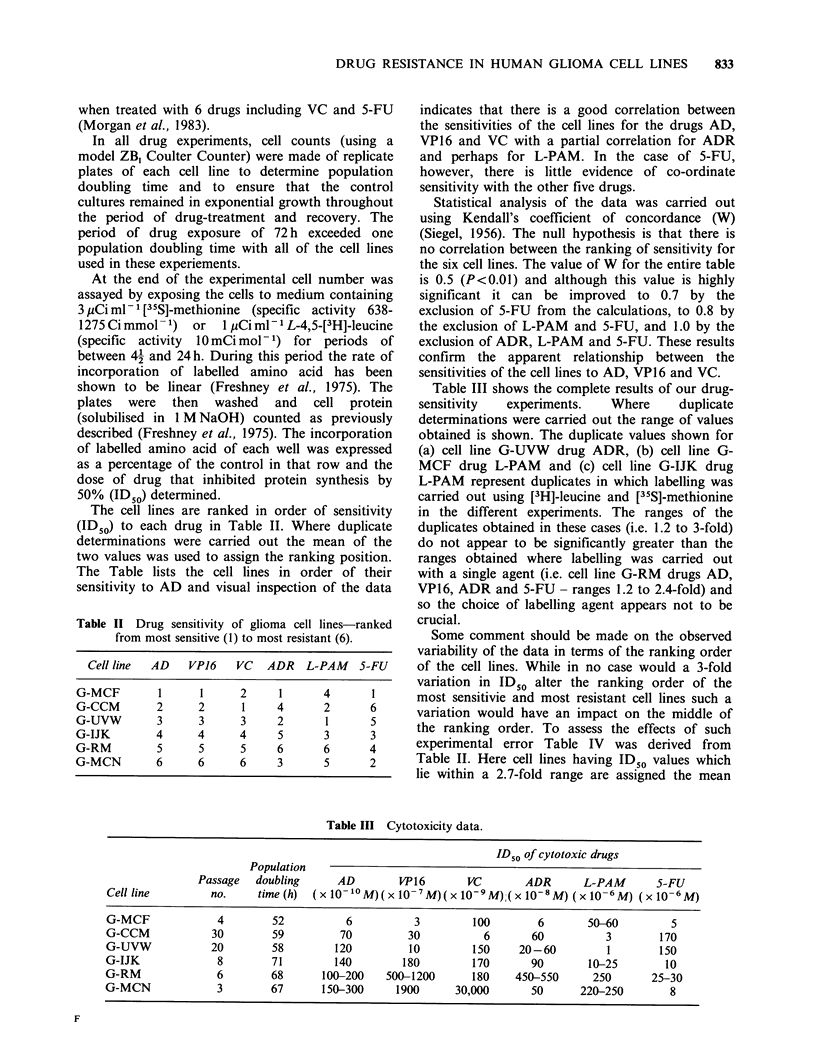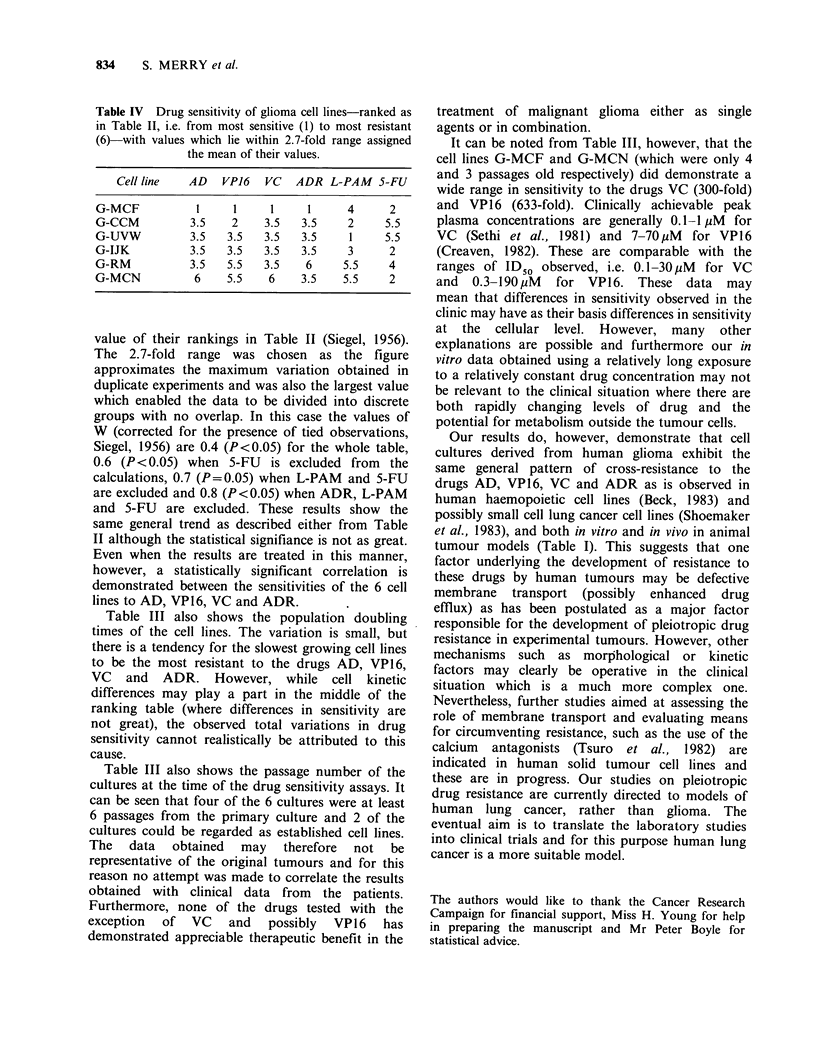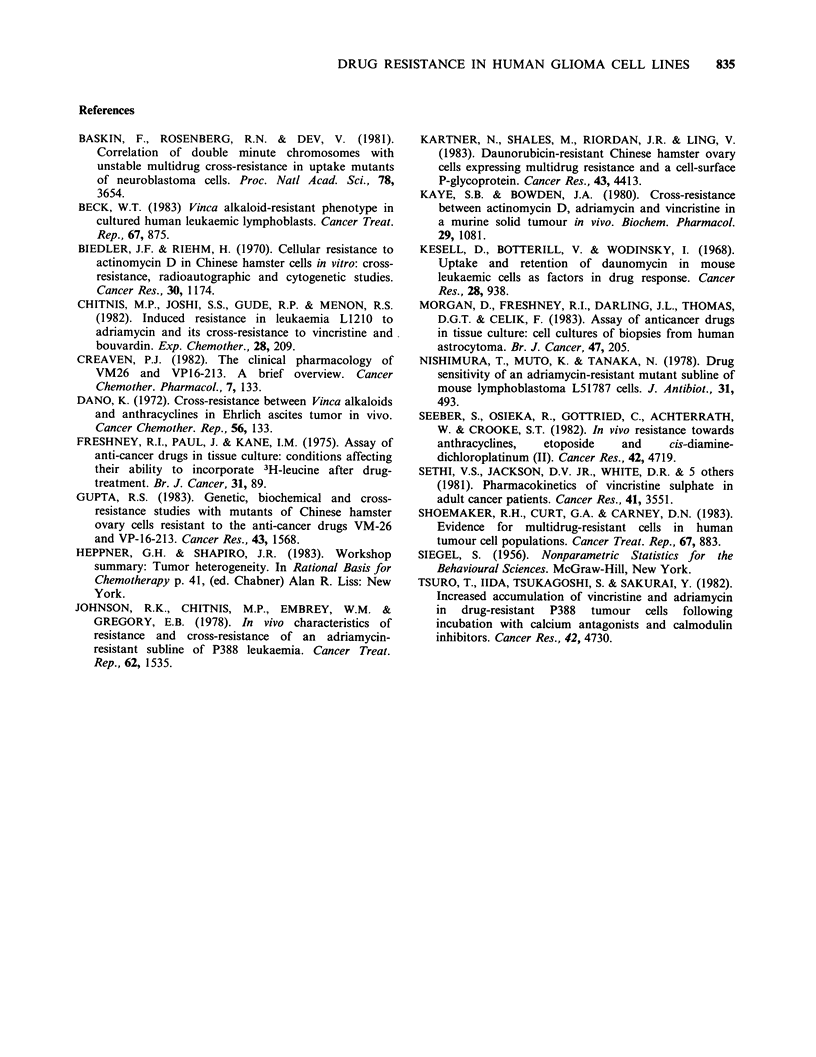# Cross-resistance to cytotoxic drugs in human glioma cell lines in culture.

**DOI:** 10.1038/bjc.1984.263

**Published:** 1984-12

**Authors:** S. Merry, S. B. Kaye, R. I. Freshney


*Br. J. Cancer* (1984), **50**, 831–835

### Short Communication

### Cross-resistance to cytotoxic drugs in human glioma cell lines in culture

S. Merry, S.B. Kaye & R.I. Freshney

*Department of Clinical Oncology, University of Glasgow, 1 Horselethill Road, Glasgow G12 9LX, UK.*

In several animal tumour models cross-resistance has been observed between the antibiotic actinomycin D (AD), the anthracyclines adriamycin (ADR) and daunorubicin (DNR), the semi-synthetic podophyllotoxin VP16-213 (VP16) and the *Vinca* alkaloids vincristine (VC) and vinblastine (VBL) (see Table I). Cross-resistance has been demonstrated both *in vitro* (Biedler & Riehm, 1970; Nishimura *et al.*, 1978; Baskin *et al.*, 1981) and *in vivo* (Kessel *et al.*, 1968; Dano, 1972; Kaye & Bowden, 1980; Chitnis *et al.*, 1982; Seeber *et al.*, 1982) and in general the cross-resistance does not extend to other drugs.

This phenomenon, known as pleiotropic drug resistance, is interesting because of the structural and biological dissimilarity of these drugs. It is possible that a common mechanism underlies the emergence of resistance to these compounds which may have important consequences in the clinical treatment of cancer using chemotherapy.

Data from human tumours are limited. There are few clinical data regarding the emergence of pleiotropic drug resistance, but it is a general impression that when resistance to a cytotoxic drug regimen develops clinically, even though it is possible to identify other regimens which have short-lived activity, universal drug resistance usually ensues. However the clinical situation is a complex one since cytotoxic drugs are generally given in combination, which makes the recognition of any particular pattern of cross resistance unlikely.

Data from human cell lines in culture are also limited and it remains unclear whether cross-resistance between antibiotics, anthracyclines, VP16-213 and *Vinca* alkaloids is a common finding *in vitro*. Beck (1983) has however demonstrated cross-resistance between *Vinca* alkaloids, DNR, VP16, AD and colchicine in a human leukaemic lymphoblast cell line originally selected for resistance to VBL. This cell line remained sensitive to podophyllotoxin, methotrexate and 6-mercaptopurine. Shoemaker *et al.* (1983) have also reported pleiotropic drug resistance in human small cell lung cancer cell lines derived from patients with progressive disease during combination chemotherapy. In this case, however, the fact that the tumours had been treated with drugs in combination means that different mechanisms of resistance to single agents may be operative. This situation is therefore possibly different from that of the animal models where treatment with a single agent has been shown to induce resistance to other structurally and functionally unrelated agents.

There is some difficulty in extrapolating results obtained from cell lines to the clinical situation. Human tumours are known to display a considerable degree of heterogeneity and there are generally different clonal subpopulations within a single tumour (Heppner & Shapiro, 1983). Thus it is conceivable that the chemosensitivity of a cell line obtained from a tumour may not be representative of the tumour as a whole. Nevertheless, the demonstration of pleiotropic drug resistance in human tumour cell lines would provide a rational basis for attempts to overcome clinically observed resistance to specific drugs, e.g. adriamycin, vinca alkaloids, using a number of agents, notably calcium antagonists (Tsuro *et al.*, 1982), which have been shown to be effective in animal and human tumour cell lines.

In this study we examine the *in vitro* sensitivity of six cell lines established from individual cases of human glioma to AD, ADR, VC, VP-16, 5-Fluorouracil (5-FU) and L-phenylalanine mustard (L-PAM). Human glioma cell lines were chosen for this study because of their relative ease of culture.

Cell lines were obtained from human glioma as described previously (Morgan *et al.*, 1983). All the tumours used were malignant astrocytomas (Kernohan grade III or IV) confirmed from paraffin wax section by Prof. D.I. Graham (Department of Neuropathology, Institute of Neurological Sciences, Southern General Hospital,

Correspondence: S. Merry
Received 31 August 1984; accepted 29 September 1984.

© The Macmillan Press Ltd., 1984

**Table I** Cross-resistance to cytotoxic drugs in animal models.

| *Author* | *Cell Line* | *AD* | *ADR/DNR* | *VP16* | *VBL/VC* |
|---|---|---|---|---|---|
| Kessel *et al.* (1968) | P815 Murine leukaemia | —** | R* | — | R |
| Biedler & Riehm (1970) | Chinese hamster transformed | R | R | — | R |
| Dano (1972) | Ehrlich ascites | — | R | — | R |
| Johnson *et al.* (1978) | P388 | R | R | R | R |
| Nishimura *et al.* (1978) | L51787 murine lymphoblastoma | — | R | — | R |
| Kaye & Bowden (1980) | ROS murine sarcoma | R | R | — | R |
| Baskin *et al.* (1981) | C46 murine neuroblastoma | — | R | — | R |
| Seber *et al.* (1982) | Ehrlich ascites | — | R | R | — |
| Chitnis *et al.* (1982) | L1210 | — | R | — | R |
| Kartner *et al.* (1983) | CH0 | — | R | — | R |
| Gupta (1983) | CH0 | R | R | R | R |

*Resistance observed. Where more than one drug appears at the top of the column R indicates resistance to either or both of the drugs.
**Drug not studied.

Glasgow, U.K.). All the cell lines grew as monolayers in culture.

The growth medium used for the cell cultures was Ham's F10 supplemented with Eagle's MEM non-essential amino acids (Flow Laboratories), 50 units $ml^{-1}$ benzyl penicillin, 50 $\mu g\,ml^{-1}$ streptomycin sulphate and with a gas phase of 5% $C0_2$. The medium was supplemented with 10% foetal bovine serum (Flow Laboratories).

The drugs used in this study were adriamycin (Farmitalia Carlo Erba Ltd, Barnet, Herts, U.K.), actinomycin D (trade name "Lyovac Cosmegen", Merk Sharp and Dohme International, Rahway, NJ, U.S.A.), vincristine sulphate (trade name "Oncovin", Eli Lilly and Co. Ltd, Basingstoke, U.K.), VP16-213 (trade name "Vepesid", Bristol Myers Pharmaceuticals, Slough, U.K.), 5-fluorouracil (Roche Products Ltd, Welwyn Garden City, U.K.) and L-phenylalanine mustard (melphalan, trade name "Alkeran", The Wellcome Foundation Ltd, London, U.K.). They were diluted according to manufacturers' instructions for injection and stored at −20°C until required (no longer than 1 month after freezing). They were then further diluted in culture medium to the required concentrations. In the case of L-PAM care was taken to ensure that these operations were carried out within 30 min because of the instability of the drug. In no case did the volume of diluent added with the drug exceed 1% of the final volume. Radiochemicals were obtained from Amersham International PLC (Amersham, Bucks, U.K.).

Drug sensitivity assays were carried out using a modifiaction of a method described previously (Morgan *et al.,* 1983). Exponentially growing cells were trypsinised and seeded into 96-well Linbro microtitration plates to give $10^3$ cells per well. After 3 days medium was removed from the plate by suction and replaced by fresh drug-free medium. After a further 24 h serial dilutions of the drugs were added to duplicate wells in the plate. Two wells of each row were left free of drugs to act as controls. The drug containing medium was replaced with fresh drug-containing medium after 24 and 48 h during a total period of exposure to drugs of 72 h. The drug-containing medium was then replaced with drug-free medium and the cells were allowed a recovery period of 5 days with medium changes after 3 and 4 days. The viability of cells remaining in each well was determined by isotopic precursor incorporation into protein as descibred below. This technique has been previously demonstrated to produce equivalent results to cloning for 5 glioma cell lines (including G-IJK)

when treated with 6 drugs including VC and 5-FU (Morgan *et al.*, 1983).

In all drug experiments, cell counts (using a model $ZB_I$ Coulter Counter) were made of replicate plates of each cell line to determine population doubling time and to ensure that the control cultures remained in exponential growth throughout the period of drug-treatment and recovery. The period of drug exposure of 72 h exceeded one population doubling time with all of the cell lines used in these experiements.

At the end of the experimental cell number was assayed by exposing the cells to medium containing $3 \mu Ci\, ml^{-1}$ [$^{35}$S]-methionine (specific activity 638-1275 Ci mmol$^{-1}$) or $1 \mu Ci\, ml^{-1}$ *L*-4,5-[$^{3}$H]-leucine (specific activity 10 mCi mol$^{-1}$) for periods of between $4\frac{1}{2}$ and 24 h. During this period the rate of incorporation of labelled amino acid has been shown to be linear (Freshney *et al.*, 1975). The plates were then washed and cell protein (solubilised in 1 M NaOH) counted as previously described (Freshney *et al.*, 1975). The incorporation of labelled amino acid of each well was expressed as a percentage of the control in that row and the dose of drug that inhibited protein synthesis by 50% ($ID_{50}$) determined.

The cell lines are ranked in order of sensitivity ($ID_{50}$) to each drug in Table II. Where duplicate determinations were carried out the mean of the two values was used to assign the ranking position. The Table lists the cell lines in order of their sensitivity to AD and visual inspection of the data indicates that there is a good correlation between the sensitivities of the cell lines for the drugs AD, VP16 and VC with a partial correlation for ADR and perhaps for L-PAM. In the case of 5-FU, however, there is little evidence of co-ordinate sensitivity with the other five drugs.

Statistical analysis of the data was carried out using Kendall's coefficient of concordance (W) (Siegel, 1956). The null hypothesis is that there is no correlation between the ranking of sensitivity for the six cell lines. The value of W for the entire table is 0.5 ($P<0.01$) and although this value is highly significant it can be improved to 0.7 by the exclusion of 5-FU from the calculations, to 0.8 by the exclusion of L-PAM and 5-FU, and 1.0 by the exclusion of ADR, L-PAM and 5-FU. These results confirm the apparent relationship between the sensitivities of the cell lines to AD, VP16 and VC.

Table III shows the complete results of our drug-sensitivity experiments. Where duplicate determinations were carried out the range of values obtained is shown. The duplicate values shown for (a) cell line G-UVW drug ADR, (b) cell line G-MCF drug L-PAM and (c) cell line G-IJK drug L-PAM represent duplicates in which labelling was carried out using [$^{3}$H]-leucine and [$^{35}$S]-methionine in the different experiments. The ranges of the duplicates obtained in these cases (i.e. 1.2 to 3-fold) do not appear to be significantly greater than the ranges obtained where labelling was carried out with a single agent (i.e. cell line G-RM drugs AD, VP16, ADR and 5-FU – ranges 1.2 to 2.4-fold) and so the choice of labelling agent appears not to be crucial.

Some comment should be made on the observed variability of the data in terms of the ranking order of the cell lines. While in no case would a 3-fold variation in $ID_{50}$ alter the ranking order of the most sensitivie and most resistant cell lines such a variation would have an impact on the middle of the ranking order. To assess the effects of such experimental error Table IV was derived from Table II. Here cell lines having $ID_{50}$ values which lie within a 2.7-fold range are assigned the mean

**Table II** Drug sensitivity of glioma cell lines—ranked from most sensitive (1) to most resistant (6).

| *Cell line* | *AD* | *VP16* | *VC* | *ADR* | *L-PAM* | *5-FU* |
|---|---|---|---|---|---|---|
| G-MCF | 1 | 1 | 2 | 1 | 4 | 1 |
| G-CCM | 2 | 2 | 1 | 4 | 2 | 6 |
| G-UVW | 3 | 3 | 3 | 2 | 1 | 5 |
| G-IJK | 4 | 4 | 4 | 5 | 3 | 3 |
| G-RM | 5 | 5 | 5 | 6 | 6 | 4 |
| G-MCN | 6 | 6 | 6 | 3 | 5 | 2 |

**Table III** Cytotoxicity data.

| | | | *$ID_{50}$ of cytotoxic drugs* | | | | | |
|---|---|---|---|---|---|---|---|---|
| *Cell line* | *Passage no.* | *Population doubling time (h)* | *AD* ($\times 10^{-10}$ *M*) | *VP16* ($\times 10^{-7}$ *M*) | *VC* ($\times 10^{-9}$ *M*) | *ADR* ($\times 10^{-8}$ *M*) | *L-PAM* ($\times 10^{-6}$ *M*) | *5-FU* ($\times 10^{-6}$ *M*) |
| G-MCF | 4 | 52 | 6 | 3 | 100 | 6 | 50–60 | 5 |
| G-CCM | 30 | 59 | 70 | 30 | 6 | 60 | 3 | 170 |
| G-UVW | 20 | 58 | 120 | 10 | 150 | 20–60 | 1 | 150 |
| G-IJK | 8 | 71 | 140 | 180 | 170 | 90 | 10–25 | 10 |
| G-RM | 6 | 68 | 100–200 | 500–1200 | 180 | 450–550 | 250 | 25–30 |
| G-MCN | 3 | 67 | 150–300 | 1900 | 30,000 | 50 | 220–250 | 8 |

**Table IV** Drug sensitivity of glioma cell lines—ranked as in Table II, i.e. from most sensitive (1) to most resistant (6)—with values which lie within 2.7-fold range assigned the mean of their values.

| *Cell line* | *AD* | *VP16* | *VC* | *ADR* | *L-PAM* | *5-FU* |
|---|---|---|---|---|---|---|
| G-MCF | 1 | 1 | 1 | 1 | 4 | 2 |
| G-CCM | 3.5 | 2 | 3.5 | 3.5 | 2 | 5.5 |
| G-UVW | 3.5 | 3.5 | 3.5 | 3.5 | 1 | 5.5 |
| G-IJK | 3.5 | 3.5 | 3.5 | 3.5 | 3 | 2 |
| G-RM | 3.5 | 5.5 | 3.5 | 6 | 5.5 | 4 |
| G-MCN | 6 | 5.5 | 6 | 3.5 | 5.5 | 2 |

value of their rankings in Table II (Siegel, 1956). The 2.7-fold range was chosen as the figure approximates the maximum variation obtained in duplicate experiments and was also the largest value which enabled the data to be divided into discrete groups with no overlap. In this case the values of W (corrected for the presence of tied observations, Siegel, 1956) are 0.4 ($P<0.05$) for the whole table, 0.6 ($P<0.05$) when 5-FU is excluded from the calculations, 0.7 ($P=0.05$) when L-PAM and 5-FU are excluded and 0.8 ($P<0.05$) when ADR, L-PAM and 5-FU are excluded. These results show the same general trend as described either from Table II although the statistical signifiance is not as great. Even when the results are treated in this manner, however, a statistically significant correlation is demonstrated between the sensitivities of the 6 cell lines to AD, VP16, VC and ADR.

Table III also shows the population doubling times of the cell lines. The variation is small, but there is a tendency for the slowest growing cell lines to be the most resistant to the drugs AD, VP16, VC and ADR. However, while cell kinetic differences may play a part in the middle of the ranking table (where differences in sensitivity are not great), the observed total variations in drug sensitivity cannot realistically be attributed to this cause.

Table III also shows the passage number of the cultures at the time of the drug sensitivity assays. It can be seen that four of the 6 cultures were at least 6 passages from the primary culture and 2 of the cultures could be regarded as established cell lines. The data obtained may therefore not be representative of the original tumours and for this reason no attempt was made to correlate the results obtained with clinical data from the patients. Furthermore, none of the drugs tested with the exception of VC and possibly VP16 has demonstrated appreciable therapeutic benefit in the treatment of malignant glioma either as single agents or in combination.

It can be noted from Table III, however, that the cell lines G-MCF and G-MCN (which were only 4 and 3 passages old respectively) did demonstrate a wide range in sensitivity to the drugs VC (300-fold) and VP16 (633-fold). Clinically achievable peak plasma concentrations are generally 0.1–1 $\mu$M for VC (Sethi *et al.*, 1981) and 7–70 $\mu$M for VP16 (Creaven, 1982). These are comparable with the ranges of $ID_{50}$ observed, i.e. 0.1–30 $\mu$M for VC and 0.3–190 $\mu$M for VP16. These data may mean that differences in sensitivity observed in the clinic may have as their basis differences in sensitivity at the cellular level. However, many other explanations are possible and furthermore our *in vitro* data obtained using a relatively long exposure to a relatively constant drug concentration may not be relevant to the clinical situation where there are both rapidly changing levels of drug and the potential for metabolism outside the tumour cells.

Our results do, however, demonstrate that cell cultures derived from human glioma exhibit the same general pattern of cross-resistance to the drugs AD, VP16, VC and ADR as is observed in human haemopoietic cell lines (Beck, 1983) and possibly small cell lung cancer cell lines (Shoemaker *et al.*, 1983), and both *in vitro* and *in vivo* in animal tumour models (Table I). This suggests that one factor underlying the development of resistance to these drugs by human tumours may be defective membrane transport (possibly enhanced drug efflux) as has been postulated as a major factor responsible for the development of pleiotropic drug resistance in experimental tumours. However, other mechanisms such as morphological or kinetic factors may clearly be operative in the clinical situation which is a much more complex one. Nevertheless, further studies aimed at assessing the role of membrane transport and evaluating means for circumventing resistance, such as the use of the calcium antagonists (Tsuro *et al.*, 1982) are indicated in human solid tumour cell lines and these are in progress. Our studies on pleiotropic drug resistance are currently directed to models of human lung cancer, rather than glioma. The eventual aim is to translate the laboratory studies into clinical trials and for this purpose human lung cancer is a more suitable model.

The authors would like to thank the Cancer Research Campaign for financial support, Miss H. Young for help in preparing the manuscript and Mr Peter Boyle for statistical advice.

### References

BASKIN, F., ROSENBERG, R.N. & DEV, V. (1981). Correlation of double minute chromosomes with unstable multidrug cross-resistance in uptake mutants of neuroblastoma cells. *Proc. Natl Acad. Sci.*, **78**, 3654.

BECK, W.T. (1983) *Vinca* alkaloid-resistant phenotype in cultured human leukaemic lymphoblasts. *Cancer Treat. Rep.*, **67**, 875.

BIEDLER, J.F. & RIEHM, H. (1970). Cellular resistance to actinomycin D in Chinese hamster cells *in vitro*: cross-resistance, radioautographic and cytogenetic studies. *Cancer Res.*, **30**, 1174.

CHITNIS, M.P., JOSHI, S.S., GUDE, R.P. & MENON, R.S. (1982). Induced resistance in leukaemia L1210 to adriamycin and its cross-resistance to vincristine and bouvardin. *Exp. Chemother.*, **28**, 209.

CREAVEN, P.J. (1982). The clinical pharmacology of VM26 and VP16-213. A brief overview. *Cancer Chemother. Pharmacol.*, **7**, 133.

DANO, K. (1972). Cross-resistance between *Vinca* alkaloids and anthracyclines in Ehrlich ascites tumor in vivo. *Cancer Chemother. Rep.*, **56**, 133.

FRESHNEY, R.I., PAUL, J. & KANE, I.M. (1975). Assay of anti-cancer drugs in tissue culture: conditions affecting their ability to incorporate $^3$H-leucine after drug-treatment. *Br. J. Cancer*, **31**, 89.

GUPTA, R.S. (1983). Genetic, biochemical and cross-resistance studies with mutants of Chinese hamster ovary cells resistant to the anti-cancer drugs VM-26 and VP-16-213. *Cancer Res.*, **43**, 1568.

HEPPNER, G.H. & SHAPIRO, J.R. (1983). Workshop summary: Tumor heterogeneity. In *Rational Basis for Chemotherapy* p. 41, (ed. Chabner) Alan R. Liss: New York.

JOHNSON, R.K., CHITNIS, M.P., EMBREY, W.M. & GREGORY, E.B. (1978). *In vivo* characteristics of resistance and cross-resistance of an adriamycin-resistant subline of P388 leukaemia. *Cancer Treat. Rep.*, **62**, 1535.

KARTNER, N., SHALES, M., RIORDAN, J.R. & LING, V. (1983). Daunorubicin-resistant Chinese hamster ovary cells expressing multidrug resistance and a cell-surface P-glycoprotein. *Cancer Res.*, **43**, 4413.

KAYE, S.B. & BOWDEN, J.A. (1980). Cross-resistance between actinomycin D, adriamycin and vincristine in a murine solid tumour *in vivo*. *Biochem. Pharmacol.* **29**, 1081.

KESELL, D., BOTTERILL, V. & WODINSKY, I. (1968). Uptake and retention of daunomycin in mouse leukaemic cells as factors in drug response. *Cancer Res.*, **28**, 938.

MORGAN, D., FRESHNEY, R.I., DARLING, J.L., THOMAS, D.G.T. & CELIK, F. (1983). Assay of anticancer drugs in tissue culture: cell cultures of biopsies from human astrocytoma. *Br. J. Cancer*, **47**, 205.

NISHIMURA, T., MUTO, K. & TANAKA, N. (1978). Drug sensitivity of an adriamycin-resistant mutant subline of mouse lymphoblastoma L51787 cells. *J. Antibiot.*, **31**, 493.

SEEBER, S., OSIEKA, R., GOTTRIED, C., ACHTERRATH, W. & CROOKE, S.T. (1982). *In vivo* resistance towards anthracyclines, etoposide and *cis*-diamine-dichloroplatinum (II). *Cancer Res.*, **42**, 4719.

SETHI, V.S., JACKSON, D.V. JR., WHITE, D.R. & 5 others (1981). Pharmacokinetics of vincristine sulphate in adult cancer patients. *Cancer Res.*, **41**, 3551.

SHOEMAKER, R.H., CURT, G.A. & CARNEY, D.N. (1983). Evidence for multidrug-resistant cells in human tumour cell populations. *Cancer Treat. Rep.*, **67**, 883.

SIEGEL, S. (1956). *Nonparametric Statistics for the Behavioural Sciences*. McGraw-Hill, New York.

TSURO, T., IIDA, TSUKAGOSHI, S. & SAKURAI, Y. (1982). Increased accumulation of vincristine and adriamycin in drug-resistant P388 tumour cells following incubation with calcium antagonists and calmodulin inhibitors. *Cancer Res.*, **42**, 4730.